# Supplementary material for: Assembly of the Mycobacterium tuberculosis type VII ESX-1 secretion system in Mycobacterium smegmatis identifies a new transcriptional activator of esx-1 genes and a novel TB vaccine
Source: Microbiol Spectr. 2025 Sep 12;13(10):e01131-25. doi: 10.1128/spectrum.01131-25 (PMC12502791; doi:10.1128/spectrum.01131-25)
Supplement: Fig. S1 and S2 — Fig. S1: Immunoblot analysis of recombinant M. smegmatis strains. Immunoblots of (A) 15 µg/well of total CF proteins and (B) 7.5 µg/well of total CL proteins from M. smegmatis::pYUB412 + pMD31, M. smegmatis::pYUB412 + pMDespACD, M. smegmatis::2F9 + pMD31 and MSX-1 after 10-, 20- and 30-hours of growth in modified 7H9 media. Fig. S2. Immunoblot analysis of MSX-1 cultured in the presence and absence of kanamycin selection pressure. Immunoblots of 10 µg/well of total CL proteins from MSX-1 grown with and without kanamycin selection pressure for 2-, 4-, 6- and 8-days in modified 7H9 media. [file spectrum.01131-25-s0001.pdf]

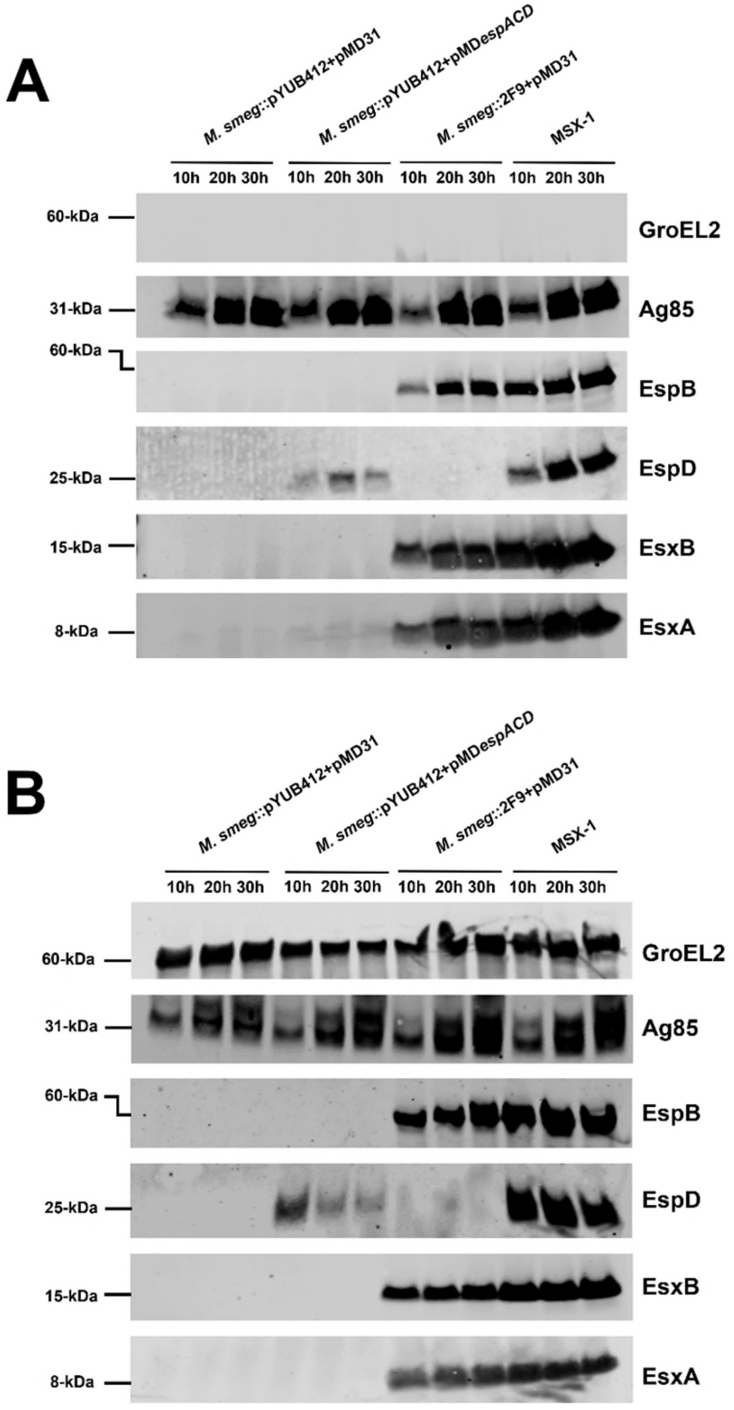

Supplementary figure 1. Immunoblot analysis of recombinant *M. smegmatis* strains. Immunoblots of (A) 15 µg/well of total CF proteins and (B) 7.5 µg/well of total CL proteins from *M. smegmatis*::pYUB412 + pMD31, *M. smegmatis*::pYUB412 + pMD*espACD*, *M. smegmatis*::2F9 + pMD31 and MSX-1 after 10-, 20- and 30-hours of growth in modified 7H9 media.

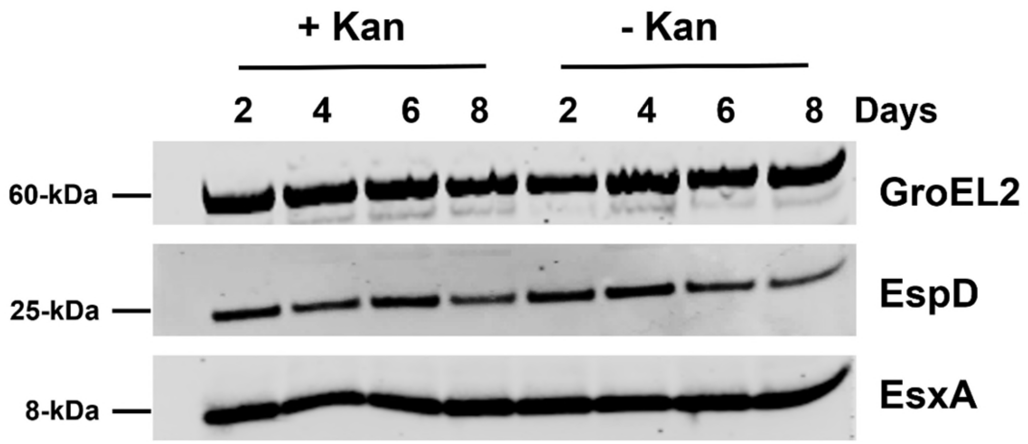

Supplementary figure 2. Immunoblot analysis of MSX-1 cultured in the presence and absence of kanamycin selection pressure. Immunoblots of 10 µg/well of total CL proteins from MSX-1 grown with and without kanamycin selection pressure for 2-, 4-, 6- and 8-days in modified 7H9 media.
